# Pharmacokinetic Comparison of 20(R)- and 20(S)-Ginsenoside Rh1 and 20(R)- and 20(S)-Ginsenoside Rg3 in Rat Plasma following Oral Administration of Radix Ginseng Rubra and Sheng-Mai-San Extracts

**DOI:** 10.1155/2017/6451963

**Published:** 2017-05-23

**Authors:** Xiaomei Fan, Yan Xu, Danni Zhu, Yibing Ji

**Affiliations:** ^1^Department of Analytical Chemistry, China Pharmaceutical University, Nanjing 210009, China; ^2^Key Laboratory of Drug Quality Control and Pharmacovigilance, Ministry of Education, Nanjing 210009, China; ^3^Department of Pharmacy, Shenzhen Bao'an Maternity and Child Health Hospital, Shenzhen 518101, China; ^4^Department of Complex Prescription of TCM, China Pharmaceutical University, Nanjing 210009, China

## Abstract

Ginsenosides Rh1 and Rg3, as the main bioactive components from Ginseng, are effective for prevention and treatment of cardiovascular diseases. Sheng-Mai-San (SMS), a classical complex prescription of traditional Chinese medicines, is composed of Radix Ginseng Rubra, Fructus Schisandrae, and Radix Ophiopogonis. In this research, a sensitive and specific liquid chromatography-mass spectrometric method was developed and validated for stereoselective determination and pharmacokinetic studies of 20(R)- and 20(S)-ginsenoside Rh1 and 20(R)- and 20(S)-ginsenoside Rg3 epimers in rat plasma after oral administration of Radix Ginseng Rubra or SMS extracts. The main pharmacokinetic parameters including *T*_max_, *C*_max_, *t*_1/2_, and AUC were calculated by noncompartment model. Compared with Radix Ginseng Rubra, SMS could significantly increase the content of ginsenosides Rh1 and Rg3 in the decocting process. Ginsenosides Rh1 and Rg3 following SMS treatment displayed higher *C*_max_, AUC_(0–t)_, and AUC_(0–*∞*)_ and longer *t*_1/2_ and *t*_max_ except for 20(R)-Rh1 in rat plasma. The results indicated SMS compound compatibility could influence the dissolution in vitro and the pharmacokinetic behaviors in vivo of ginsenosides Rh1 and Rg3, suggesting pharmacokinetic drug-drug interactions between ginsenosides Rh1 and Rg3 and other ingredients from Fructus Schisandrae and Radix Ophiopogonis. This study would provide valuable information for drug development and clinical application of SMS.

## 1. Introduction

Ginsenosides are glycosides with a dammarane skeleton and classified as protopanaxadiol or protopanaxatriol compounds (except ginsenoside Ro). Ginsenosides are the major bioactive ingredients of Ginseng and exhibit many pharmacological activities including vasorelaxation, antioxidation, anti-inflammation, and anticancer [1]. Ginseng is often prescribed in combinations with other herbs to obtain better therapeutic potentials.

**Figure 1:**
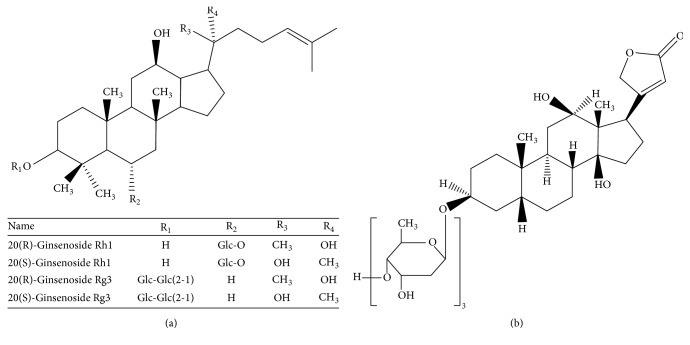
Chemical structures of ginsenosides Rh1 and Rg3 (a) and digoxin (b).

Sheng-Mai-San (SMS), a classical complex prescription of traditional Chinese medicine (TCM), contains Radix Ginseng Rubra, Fructus Schisandrae, and Radix Ophiopogonis [2]. It was found that SMS possessed extensive biological and pharmacological activities, such as cardiovascular activities, antioxidative activities, and protective effects on tissue injury [3, 4]. SMS could protect against renal ischaemic damage during heat stroke by reducing iNOS-dependent NO and peroxynitrite production [5, 6]. Besides these, it could reduce hepatic lipids and lipid peroxidation [7]. Synergetic effects and interactions of multiple constituents usually exist in compound prescriptions. The absorption, distribution, metabolism, and excretion of active ingredients in herbs are often influenced by other herbs [8, 9]. Pharmacokinetic study is an effective strategy to understand the mechanism of drug-drug interactions in clinical application [10, 11]. Ginsenosides Rh1 and Rg3 (Figure 1), which are both racemic mixture of 20(R)- and 20(S)-epimers, are the main bioactive ginsenosides contained in SMS. Pharmacokinetic studies of the racemate [12, 13] or single epimer [14] of ginsenosides Rh1 and Rg3 were widely done; however, simultaneous determination and pharmacokinetic study of their two epimers in rat plasma was seldom reported. Whether SMS compound compatibility affects the contents and the pharmacokinetic behaviors of ginsenosides Rh1 and Rg3 remains unclear. The aim of this research is to investigate the possible pharmacokinetic differences of 20(R)- and 20(S)-ginsenoside Rh1 and 20(R)- and 20(S)-ginsenoside Rg3 in rats after oral administration of single Radix Ginseng Rubra and SMS extracts and to explore herb-herb interaction on ginsenosides of SMS in vitro and in vivo.

## 2. Materials and Methods

### 2.1. Chemicals and Regents

Radix Ginseng Rubra was from Jilin province in China. Radix Ophiopogonis was purchased from Mianyang of Sichuan province. Fructus Schisandrae was from Liaoning province. The three herbs were authenticated by Professor Min-jian Qin (Department of Resources Science of Traditional Chinese Medicine, China Pharmaceutical University). Digoxin (internal standard, purity > 98%) was purchased from the National Institute for the Control of Pharmaceutical and Biological Products (Beijing, China). 20(R)- and 20(S)-ginsenoside Rh1 and 20(R)- and 20 (S)-ginsenoside Rg3 (purity > 98%) were from Nanjing Zelang Medical Technological Co. Ltd. (Nanjing, China). D101 macroporous resin was provided by Chemical Plant of Nankai University (Tianjin, China). Ammonium chloride, ammonium acetate, and acetic acid were purchased from Nanjing Reagent Company (Nanjing, China). Acetonitrile (Tedia, USA) and methanol (Hanbang, China) were of HPLC grade.

### 2.2. LC-MS Analysis

A Shimadzu 2020 LC-MS system, with an electrospray ionization interface (ESI) source and Shimadzu LCMS LabSolution Workstation software for data processing, was utilized to perform the analytical procedures. The system includes two Shimadzu LC-20AD pumps, a Shimadzu SIL-20A autosampler, a Shimadzu CTO-20A column oven, a CBM-20A system controller, and a Shimadzu DGU-20A3 online degasser. A Q-array-octapole-quadrupole mass analyzer was used as the detector.

The chromatographic separation was carried on a Diamonsil C_18_ column (150 mm × 4.6 mm id, 5 *μ*m; Dikma, China) with the mobile phase containing 0.5 mmol/L ammonium chloride in double-distilled water (A) and acetonitrile (B) at a flow rate of 1.0 mL/min. A diverter valve was used to direct 0.3 mL/min HPLC elute to MS detector and 0.7 mL/min elute to a waste container. A gradient elution program was used as follows: 0.01→6.0 min, 38% B; 6.0→6.5 min, 38→50% B; 6.5→15.0 min, 50% B; 15.0→15.5 min, 50→38% B. The column temperature was kept constant at 40°C.

The negative-ion electrospray ionization mode was utilized under the following source conditions: curved desolvation line (CDL) temperature, 250°C; block temperature, 400°C; interface temperature, 350°C; nebulizing gas flow rate, 1.5 L/min; drying gas flow rate, 10 L/min. CDL voltage, 0 V; Q-array DC (direct current) voltage, 0 V; interface voltage and RF (radiation frequency) voltage were fixed as those in tuning. The chlorinated adducts of molecular ions [M + Cl]^−^ of 20(R)-ginsenoside Rh1 and 20(S)-ginsenoside Rh1 at* m/z* 673.45, 20(R)-ginsenoside Rg3 and 20(S)-ginsenoside Rg3 at* m/z* 819.60, and digoxin (IS) at* m/z* 815.60 were monitored in selected ion monitoring (SIM) mode.

### 2.3. Preparation of SMS and Radix Ginseng Rubra Extracts

Radix Ginseng Rubra and Radix Ophiopogonis were cut into slices and Fructus Schisandrae was scrunched into powder before use. Three drugs were mixed in the ratio of 1 : 3 : 1.5 and the total weight was 110 g [15]. The mixture was extracted with water for three times (1 : 10, 1 : 8, and 1 : 6, w/v) at 100°C, 1 h for each time. The SMS extract was obtained and condensed to 0.55 g/mL crude drug. 20 g Radix Ginseng Rubra was treated as above and the extract was condensed to 0.1 g/mL crude drug. SMS and Radix Ginseng Rubra concentrated solution was subjected to D101 macroporous resin and eluted with water followed by 95% ethanol. The 95% ethanol eluate was further concentrated to 5.5 g/mL crude drug for SMS and 1.0 g/mL crude drug for Radix Ginseng Rubra before use.

### 2.4. Preparation of Stock Solutions, Calibration Samples, and Quality Control Samples

The stock solutions of 20(R)-Rh1, 20(S)-Rh1, 20(R)-Rg3, and 20(S)-Rg3 were prepared in methanol and serially diluted with methanol to prepare working solutions. A 100 ng/mL internal standard (IS) solution was similarly prepared by diluting 0.2 mg/mL stock solution of digoxin in methanol. All solutions were passed through 0.45 *μ*m membrane filter and stored at 4°C. Calibration standards of 20(R)-Rh1, 20(S)-Rh1, 20(R)-Rg3, and 20(S)-Rg3 (0.5, 1.0, 5.0, 10.0, 25.0, 50.0, 75.0, and 100 ng/mL, resp.) were obtained by spiking appropriate amount of working solutions to blank plasma. For quality control (QC) samples, four ginsenosides were prepared at concentrations of 1.0, 10.0, and 90.0 ng/mL in plasma, respectively. The standard spiked plasma samples were stored at −20°C until analysis.

### 2.5. Sample Preparation

An aliquot of 300 *μ*L plasma sample was transferred into a centrifuge tube together with 10 *μ*L of IS solution. After vortex-mixing for 30 s, the analytes and IS were extracted from plasma with *n*-butanol for three times (1 : 3, 1 : 2, and 1 : 2, v/v). Then the organic layer was transferred into another centrifuge tube and evaporated to dryness at 37°C. Finally, the residue was reconstituted in 20 *μ*L methanol and centrifuged at 12,000 ×g for 10 min; 10 *μ*L aliquot was injected into chromatographic system for analysis.

### 2.6. Pharmacokinetic Study

Male Sprague-Dawley rats, 193–215 g in weight, were supplied by Shanghai Slater Experimental Animal Feeding Center (Shanghai, China). The rats were kept in an air-conditioned animal quarter at a temperature of 22 ± 2°C and a relative humidity of 50 ± 10%. The animals were acclimatized to the facilities for four days and then fasted with free access to water for 12 h prior to experiment. Animal experiments were carried out in accordance with the Regulations of Experimental Animal Administration issued by the Ministry of Science and Technology of China. All animal protocols were approved by the Ethics Committee on the Care and Use of Laboratory Animals of China pharmaceutical University.

Rats were divided randomly into two groups (5 rats in each time point per group) and given an oral dose of 1.35 mL/100 g Radix Ginseng Rubra and SMS extracts, respectively, which was derived from the dose in clinical practice with correction. About 0.5 *μ*L blood samples were collected in heparinized Eppendorf tube via the oculi chorioideae vein before dosing and subsequently at 0.083, 0.25, 0.5, 0.75, 1, 1.5, 2, 4, 6, 8, and 12 h following oral administration of Radix Ginseng Rubra or SMS extracts. After being mixed gently and centrifuged at 3,000 ×g for 10 min, the plasma sample was obtained and kept at −20°C until analysis.

### 2.7. Method Validation

The method was validated by linearity, the lower limit of quantification (LLOQ), intra- and interday precision, accuracy, extraction recovery, and stability. Plasma samples were quantified by using the ratio of the peak area of analyte to that of IS as the assay parameter. Standard curves representing peak area ratios versus analyte concentrations were described in the form of *y* = *ax* + *b*. For the sensitivity determination, a series of different diluted plasma standard samples were prepared. The LLOQ was estimated using a signal-to-noise ratio of 10 : 1. Intra- and interday precision and accuracy were assayed by determining QC samples at high, middle, and low concentration levels on three different validation days. The extraction recoveries of analytes at three QC levels were evaluated by comparing the peak areas obtained from QC samples with those from the standard solutions at the same concentration. The stability of analytes in rat plasma was investigated by analyzing QC samples at three concentrations stored for 12 h at ambient temperatures and after three freeze (−20°C) thaw (room temperature) cycles. The matrix effect was determined by comparing the peak response ratio of each analyte in blank plasma spiked with QC working solutions at three levels with that of each analyte in the corresponding concentration of prepared QC working solutions using pure water instead of blank plasma. For each concentration level, three duplicates were operated.

### 2.8. Data Analysis

Data are expressed as the mean ± standard deviation (SD). Pharmacokinetic parameters were estimated by noncompartment model using WinNonlin 5.3 program package (Pharsight Corporation, Mountain View, CA). The area under the curve (AUC_0–t_) was calculated using the linear-trapezoidal rule, with extrapolation to infinity (AUC_0–*∞*_) from the last detectable concentration using the terminal elimination rate constant (*k*_*e*_) calculated by linear regression of the final log-linear part of the drug concentration-time curve. Apparent elimination half-life (*t*_1/2_) was calculated as *t*_1/2_ = 0.693/*k*_*e*_. Ginsenoside AUC_C_ value is derived from AUC_0–*∞*_/concentration ratio (SMS/Radix Ginseng Rubra). Differences between groups were evaluated by unpaired Student's *t*-test. Differences were considered statistically significant at *P* < 0.05.

## 3. Results

### 3.1. Optimization of Chromatographic Conditions

Owing to the similarity of chemical structure, retention action, extraction efficiency, and ionization with ginsenosides, digoxin was adopted as the internal standard in the present study. The MS responses of 20(R)-Rh1, 20(S)-Rh1, 20(R)-Rg3, 20(S)-Rg3, and digoxin to ESI were evaluated by measuring the full scan mass spectra in both positive and negative ionization modes. To obtain the maximum sensitivity, we investigated the effects of pH and additives with various mobile phases on the ionization efficiency of analytes. Compared with ammonium acetate and acetic acid, ammonium chloride as mobile phase additive showed the maximum sensitivity of 20(R)-Rh1, 20(S)-Rh1, 20(R)-Rg3, 20(S)-Rg3, and digoxin in negative mode.

### 3.2. Selection of the Plasma Sample Pretreatment Method

Various plasma sample pretreatment procedures were evaluated, including protein precipitation and liquid-liquid extraction (LLE). Conventional and simple liquid-liquid extraction procedure with *n*-butanol displayed less interference and thus was selected for sample pretreatment in our study. In addition, in view of the trace concentration of ginsenosides Rh1 and Rg3 in rat plasma following oral administration of SMS and Radix Ginseng Rubra extracts, only applying HPLC-MS method could not meet the analysis requirement in this study. For the plasma samples, concentrating the extract of LLE is another good choice for raising the concentration of analytes. In our study, 300 *μ*L sample plasma was liquid-liquid extracted by *n*-butanol, evaporated to dryness, and then reconstituted by 25 *μ*L methanol. The concentration of the analytes was condensed 12-fold around, and the sensitivity of the method was obviously improved.

### 3.3. Method Validation

**Figure 2:**
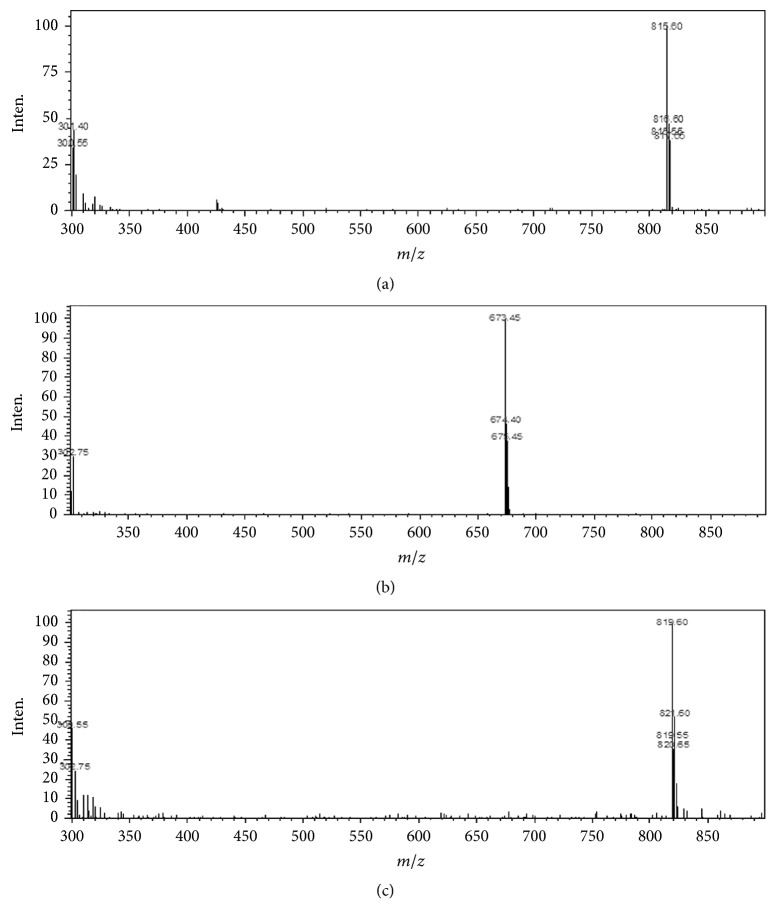
Negative-ion electrospray mass spectra obtained in scan mode from authentic samples of digoxin (a), ginsenoside Rh1 (b), and ginsenoside Rg3 (c) with abundance of [M + Cl]^−^.

**Figure 3:**
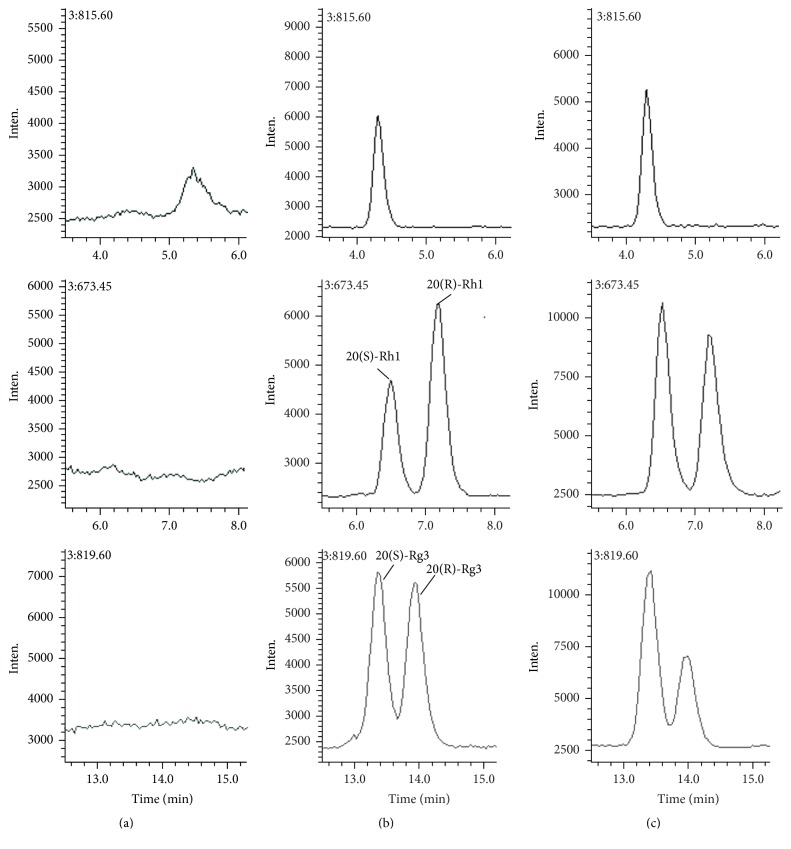
SIM chromatograms of blank plasma (a), blank plasma spiked with 25 ng/mL of 20(R)-Rh1, 20(S)-Rh1, 20(R)-Rg3, 20(S)-Rg3, and 20 ng/mL of internal standard (b), plasma sample from rat at 0.0833 h after oral administration of SMS extract (c).* m/z* 815.60, 673.45, and 819.60 for digoxin, 20(R,S)-Rh1, and 20(R,S)-Rg3, respectively.

The full scan mass spectra of Rh1 and Rg3 after direct injection in mobile phase are presented in Figure 2. Protonated molecules [M − H]^−^ of Rh1, Rg3, and digoxin were not detected. The predominant protonated molecules found for Rh1, Rg3, and digoxin were [M + Cl]^−^* m/z* 673.45, 819.60, and 815.60, respectively. The mass spectrometric parameters were optimized to obtain the higher signal for the selected ion [M + Cl]^−^, which also showed less internal interference. The typical chromatograms of 20(R)-Rh1, 20(S)-Rh1, 20(R)-Rg3, and 20(S)-Rg3 were presented in Figure 3. Under the described chromatographic conditions, a good separation was achieved and no obvious interference from endogenous plasma substances was observed. This indicated that the specificity of the method procedure was appropriate. The retention time of 20(S)-Rh1, 20(R)-Rh1, 20(S)-Rg3, 20(R)-Rg3, and IS was 6.5, 7.2, 13.4, 13.9, and 4.3 min, respectively.

The linear regression analysis of analytes was constructed by plotting the ratio of peak area of analytes to that of IS (*y*) versus analytes concentration in spiked plasma samples (*x*). The linear range and regression equations for quantification of 20(R)-Rh1, 20(S)-Rh1, 20(R)-Rg3, and 20(S)-Rg3 were presented in Table 1. The correlation coefficients of these calibration curves were all higher than 0.9935. The lower limits of quantification (LLOQ) of these analytes were 0.5~1.0 ng/mL. In comparison to some methods reported using MS detector [14, 16], a higher sensitivity for 20(R)-Rh1, 20(S)-Rh1, 20(R)-Rg3, and 20(S)-Rg3 was obtained in our study, and thus the method developed showed more advantages to determine the trace concentration of these analytes in plasma.

The extraction recoveries of 20(R)-Rh1, 20(S)-Rh1, 20(R)-Rg3, and 20(S)-Rg3 in rat plasma were shown in Table 2. At three concentration levels of these analytes, the extraction recoveries were all more than 70%, and the RSD for the intra- and interday precision was all less than 11.7%. The mean accuracy of the analytes was within the range of 89.2~109.5%. For stability test, the RSD of four ginsenosides was no more than 12%, which indicated that these analytes in rat plasma were stable for three cycles of freeze-thaw and 12 h at room temperature. Additionally, apparent matrix effect was not found to affect assay precision for four ginsenosides and internal standard. The mean matrix effects of 20(R)-Rh1, 20(S)-Rh1, 20(R)-Rg3, 20(S)-Rg3, and digoxin were all less than 106.2% (ranged from 94.1% to 117.5% for different QC levels) plasma sample.

### 3.4. Content of Four Ginsenosides in Radix Ginseng Rubra and SMS Extracts

The contents of ginsenosides Rh1 and Rg3 in Radix Ginseng Rubra and SMS extracts were determined under the above chromatographic conditions. About 1 mL of herbal extract was diluted 5 times with water, and then 1 mL of the diluent was dissolved in 4 mL methanol and ultrasonicated for 30 min. The solution was filtered through 0.45 *μ*m nylon membrane filter before injection. The results are given in Table 3. Compared with Radix Ginseng Rubra extract, a higher content of 20(R)-Rh1, 20(R)-Rg3, and 20(S)-Rg3 and a low content of 20(S)-Rh1 in decoction are obtained for SMS when using the same amount of Radix Ginseng Rubra. The ratio of ginsenoside contents in SMS to that from Radix Ginseng Rubra was 1.39 for 20(R)-Rh1, 0.62 for 20(S)-Rh1, 3.04 for 20(R)-Rg3, and 1.50 for 20(R)-Rg3. It was indicated that some ingredients in Fructus Schisandrae and Radix Ophiopogonis could increase the dissolution of 20(R)-Rh1, 20(R)-Rg3, and 20(S)-Rg3, and the opposite for 20(S)-Rh1 in the decocting process.

### 3.5. Plasma Drug Concentration-Time Curve

**Figure 4:**
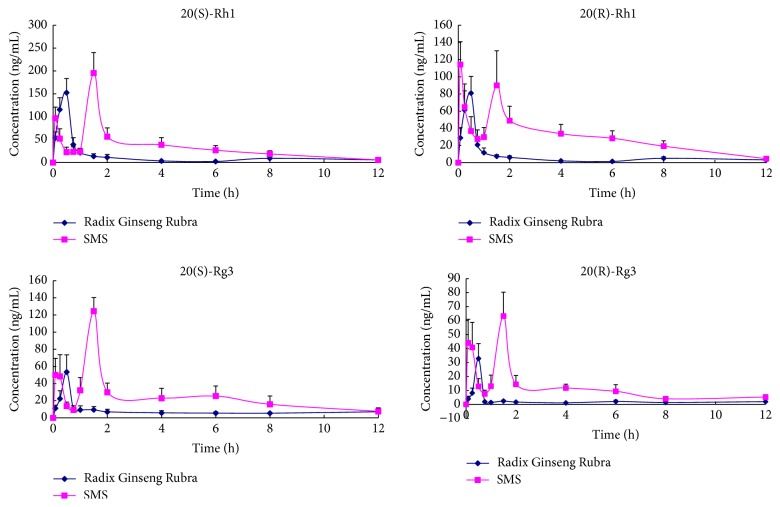
Mean plasma concentration-time profiles of 20(R)-Rh1, 20(S)-Rh1, 20(R)-Rg3, and 20(S)-Rg3 in rats after oral administration of Radix Ginseng Rubra (*◆*) and SMS (■) extracts.

The plasma concentrations of 20(R)-Rh1, 20(S)-Rh1, 20(R)-Rg3, and 20(S)-Rg3 in rats were determined successfully following oral administration of Radix Ginseng Rubra and SMS extracts. The administration doses of four ginsenosides from SMS and Radix Ginseng Rubra extracts were different in this study. AUC value of each ginsenoside was converted to AUCc according to their doses due to AUC value proportional to dose for linear pharmacokinetics [17]. Main pharmacokinetic parameters are presented in Table 4. The plasma concentration-time curves are illustrated in Figure 4. From the figure and table, there are significant differences in the pharmacokinetic process of four ginsenosides investigated between SMS and Radix Ginseng Rubra. The pharmacokinetic parameters of four ginsenosides after oral administration of SMS, in comparison with Radix Ginseng Rubra alone, show a higher *C*_max_, AUC_(0–t)_, and AUC_(0–*∞*)_ and longer *t*_1/2_ and *t*_max_ except for 20(R)-Rh1. The significant increase in AUC and *C*_max_ indicates that ginsenosides Rh1 and Rg3 were absorbed better in the intragastric administration of SMS than the Radix Ginseng Rubra extract. In addition, long *t*_1/2_ and *t*_max_ infer a delayed absorption and slow elimination of four ginsenosides after oral administration of SMS. It suggests that compound compatibility of SMS, that is, Radix Ginseng Rubra combined with Fructus Schisandrae and Radix Ophiopogonis, would affect the pharmacokinetics of ginsenosides Rh1 and Rg3 including promoting the absorption in vivo, inhabiting the elimination, and improving the bioavailability. However, the pharmacokinetic behavior and parameters of 20(R)-Rh1, 20(S)-Rh1, 20(R)-Rg3, and 20(S)-Rg3 were different from the literature reported [12, 18]. This can be explained by the different dosage and sample solution, the compound compatibility, and the administration way. The pharmacokinetic results indicate that some ingredients in the other two herbs of SMS could effectively influence the pharmacokinetic behavior of ginsenosides Rh1 and Rg3 in rats. The current studies emphasize the significance of the research toward an understanding of pharmacokinetic interaction and synergetic effect of the coexisting components in the complex prescription.

## 4. Discussion

TCM has been applied in clinical practice for more than 2000 years. Complex herbal prescriptions are the commonly used forms of TCM. Unlike chemically synthetic drugs of high purity, herbal medicines either as single herb or as combination of two or more herbs in composite formula contain hundreds of phytochemicals. However, only the constituents absorbed to blood and their metabolites possess the probability to exert pharmacological activities through the oral administration way. Generally, the compound compatibility of TCM influences the absorption, distribution, metabolism, and excretion in vivo of active ingredients [8, 9]. Synergetic effects and interactions of multiple constituents exist in the complex prescription, and some ingredients considered to be inactive could promote the absorption of effective constituents to blood [19].

Ginsenosides are regarded as the principal ingredients responsible for the pharmacological activities of SMS. However, pharmacokinetic studies of ginsenosides are challenging, especially for ginsenosides epimers. Firstly, because of the UV end absorption of ginsenosides, HPLC-UV method detection performed at 200~205 nm usually caused high level of baseline noise. Secondly, the amount of ginsenosides in SMS is relatively low. Furthermore, the weak bioavailability of ginsenosides following oral administration results in trace plasma concentration [18]. These suggest that a sensitive and specific method is required for the determination and pharmacokinetic study of ginsenosides absorbed to the blood in SMS. LC-MS method has been widely used for ginsenoside analysis [20–23].

Ginsenosides Rh1 and Rg3 are main bioactive components of Radix Ginseng Rubra and confirmed as main effective constitutes in some compound prescriptions of TCM, such as Sheng-Mai-San. Ginseng-herb interactions were previously reported [24–26]. Exploring potential drug-drug interactions in vitro and in vivo would be of great help to elucidate compatibility of various components in herbs and compound prescriptions, which might provide guideline for better clinical application. In this study, we found that interactions between ginsenosides Rh1 and Rg3 and other components from Fructus Schisandrae and Radix Ophiopogonis existed. The concentration of 20(R)-Rg3, 20(S)-Rg3, and 20(R)-Rh1 in SMS decoction was significantly increased except for 20(S)-Rh1 compared with those in Radix Ginseng Rubra extracts, although they had the same quantity of Radix Ginseng Rubra (crude drug). It suggested that there might be some solubilizing agents in Fructus Schisandrae, Radix Ophiopogonis, or both of them, which could increase the contents of ginsenosides in SMS extract. Previous studies indicated that the types and contents of ginsenosides changed during the extraction process of SMS. Coextraction with Fructus Schisandrae and Radix Ophiopogonis significantly decreased the contents of Rb1, Rb2, Rc, Rd, Re, and Rg1 from Radix Ginseng Rubra but increased Rg3 and Rh1 contents. With compound compatibility between Radix Ginseng Rubra and Fructus Schisandrae, some ginsenosides disappeared and the total contents of ginsenosides were only 25% of that from single Radix Ginseng Rubra extract [15]. Solubilization, salification, complexation, oxidation, hydrolytic decomposition, and reduction usually occurred when decocting with other herbs. How the content of chemical ingredients in herbs changed in decocting progress was always studied [15, 27, 28], which could help elucidate the underlying mechanism of compatibility of traditional Chinese medicine in vitro. This study revealed that dissolution of ginsenosides in Radix Ginseng Rubra would change when decocted with Fructus Schisandrae and Radix Ophiopogonis.

In pharmacokinetic study, ginsenosides Rh1 and Rg3 in Radix Ginseng Rubra showed quick elimination in vivo with short *t*_1/2_ of 2.94 ± 0.76 h, 2.85 ± 0.69 h, 3.70 ± 0.43 h, and 4.14 ± 1.18 h for 20(R)-Rh1, 20(S)-Rh1, 20(R)-Rg3, and 20(S)-Rg3, respectively. The elimination of ginsenoside Rh1 was significantly postponed (*t*_1/2_ increased to 3.69 ± 0.87 h for 20(R)-Rh1, 4.21 ± 1.05 h for 20(S)-Rh1, 4.45 ± 0.98 h for 20(R)-Rg3, and 6.38 ± 1.72 h for 20(S)-Rg3) when it was coadministrated with Fructus Schisandrae and Radix Ophiopogonis. Furthermore, coadministration with SMS extracts markedly increased the systemic exposure level of 20(R)-Rh1, 20(S)-Rh1, 20(R)-Rg3, and 20(S)-Rg3 in vivo with AUC_c_ 86.90→271.76, 159.20→677.02, 29.50→53.82, and 95.00→212.87, respectively. Double peak phenomenon was observed in rats following oral administration of SMS extract compared with those in Radix Ginseng Rubra, which might be caused by hepatoenteral circulation, transformation from other ginsenosides, or other reasons. Under catalysis by gastric acid and intestinal bacteria in biological conditions, both protopanaxadiol- and protopanaxatriol-type ginsenosides undergo sequential deglycosylation metabolism to form secondary metabolites and finally aglycones [29]. Regulating liver microsomal cytochrome P450-mediated drug metabolism might be responsible for pharmacokinetic drug-drug interactions. It was reported that protopanaxatriol ginsenosides including Rh1 mainly metabolized by CYP3A and (or) CYP2D were identified as the substrates of these enzymes [21]. Other components from Fructus Schisandrae or Radix Ophiopogonis may also exert competitive inhibition of other CYP3A4 substrates. Our previous study indicated that extract of Fructus Schisandrae and lignans exhibited inhibitory effect on liver microsomal CYP3A4 and CYP2D6 in vitro [30–32]. It suggests that Fructus Schisandrae may inhibit CYP3A4-catalyzed oxygenation metabolism and further alleviate systematic clearance of PPT-type ginsenosides. In addition, schisandra lignan was found to be a strong P-glycoprotein (P-gp) inhibitor, which indicated a potential herb-drug interaction to increase the absorption of P-gp substrate drugs when schisandra lignan was coadministered with these drugs [33, 34]. It was reported that schisandra lignans could significantly enhance the exposure of ginsenosides Rb2, Rc, Rg2, Rg3, Rd, and Rb1 in vitro and in vivo [35, 36]. Furthermore, pharmacokinetic and pharmacodynamic drug-drug interactions between ginsenosides and schisandrin exist [17]. These previous studies would provide novel insight into the understanding of the synergistic mechanism of SMS. Additionally, the pharmacokinetic drug-drug interaction between ginsenosides and the constituents from Radix Ophiopogonis was not reported and still remained unclear. To investigate the involved mechanisms of pharmacokinetic behaviors for ginsenosides after compound compatibility, herb pairs including Radix Ginseng Rubra-Radix Ophiopogonis and Radix Ginseng Rubra-Fructus Schisandrae will be used for further research.

## 5. Conclusion

In the present study, a rapid, sensitive, and specific LC-MS was developed and validated for stereoselective determination and pharmacokinetic studies of 20(R)- and 20(S)-ginsenoside Rh1 and 20(R)- and 20(S)-ginsenoside Rg3 epimers in rat plasma after oral administration of Radix Ginseng Rubra or SMS extracts. A simple liquid-liquid extraction procedure was used to prepare the samples with high recovery. With respect to the mobile phase, 0.5 mmol/L ammonium chloride in water was preferred to obtain abundant and stable signals. The established assay was applied to the pharmacokinetic study of compound compatibility of SMS. Compared with Radix Ginseng Rubra, SMS could significantly increase the dissolution of ginsenosides Rh1 and Rg3 in the decocting process. Ginsenosides Rh1 and Rg3 following SMS treatment displayed higher *C*_max_, AUC_(0–t)_, and AUC_(0–*∞*)_ and longer *t*_1/2_ and *t*_max_ except for 20(R)-Rh1 in rat plasma. The results indicated SMS compound compatibility could influence the dissolution in vitro and the pharmacokinetic behavior in vivo of ginsenosides Rh1 and Rg3, involving increasing the absorption, inhibiting the elimination, and improving the bioavailability, which suggested pharmacokinetic drug-drug interactions between ginsenosides Rh1 and Rg3 and other ingredients from Fructus Schisandrae and Radix Ophiopogonis in SMS. This study would provide valuable information for drug development and clinical application of SMS.

## Figures and Tables

**Table 1:** The linearity of the assay for 20(R)-Rh1, 20(S)-Rh1, 20(R)-Rg3, and 20(S)-Rg3.

Analytes	Calibration curves (*y* = *ax* + *b*)	Linear range (ng/mL)	*R* ^2^
20(R)-Rh_1_	*y* = 0.0612*x* + 0.0638	0.5–95.0	0.9986
20(S)-Rh_1_	*y* = 0.0304*x* + 0.1010	0.5–99.2	0.9935
20(R)-Rg_3_	*y* = 0.0574*x* + 0.0468	0.5–99.2	0.9990
20(S)-Rg_3_	*y* = 0.0581*x* + 0.0867	0.5–100.0	0.9947

**Table 2:** The extraction recovery, precision, and accuracy of 20(R)-Rh1, 20(S)-Rh1, 20(R)-Rg3, and 20(S)-Rg3 in rat plasma (*n* = 5).

Analytes	Spiked concentration (ng/mL)	Recovery (%)	RSD (%)	Intraday		Interday	
				Precision RSD (%)	Accuracy (%)	Precision RSD (%)	Accuracy (%)
20(R)-Rh1	1.0	81.0	9.6	3.9	105.7	5.3	104.2
	10.0	82.4	2.3	3.3	96.2	4.0	101.3
	90.0	80.9	6.6	2.1	104.5	3.7	99.6

20(S)-Rh1	1.0	85.1	7.9	5.6	94.9	11.7	95.0
	10.0	81.3	3.7	5.2	105.4	5.9	106.1
	90.0	81.2	5.9	8.4	102.1	3.1	103.3

20(R)-Rg3	1.0	83.3	9.2	7.2	109.5	6.7	107.6
	10.0	70.4	3.1	4.4	103.9	6.1	102.6
	90.0	70.9	8.3	3.3	102.7	10.3	98.7

20(S)-Rg3	1.0	83.8	2.0	2.5	89.2	8.7	92.4
	10.0	71.7	2.1	7.1	96.7	7.5	95.9
	90.0	72.6	6.8	6.1	97.4	9.8	94.8

**Table 3:** Content of ginsenosides in SMS and Radix Ginseng Rubra extracts.

		Ginsenosides			
		20(R)-Rh1	20(S)-Rh1	20(R)-Rg3	20(S)-Rg3
Concentration (*μ*g/mL)	SMS (A)	180.5	240.3	140.5	134.0
	Radix Ginseng Rubra (B)	129.8	385.0	46.3	89.5
	Ratio (A/B)	1.39	0.62	3.04	1.50

**Table 4:** Pharmacokinetic parameters of 20(R)-Rh1, 20(S)-Rh1, 20(R)-Rg3, and 20(S)-Rg3 in male rats after oral administration of SMS and Radix Ginseng Rubra extracts (1.35 mL/100 g).

Herb extract	Pharmacokinetic parameters	Ginsenosides			
		20(R)-Rh1	20(S)-Rh1	20(R)-Rg3	20(S)-Rg3
SMS	*T*_max_ (h)	0.0833	1.50	1.50	1.50
	*C*_max_ (ng/mL)	114.34 ± 26.51	195.34 ± 44.91	63.26 ± 16.99^*∗∗*^	124.43 ± 15.82^*∗∗*^
	*t*_1/2_ (h)	3.69 ± 0.87	4.21 ± 1.05^*∗*^	4.45 ± 0.98	6.38 ± 1.72^*∗*^
	AUC_(0–*t*)_ (ng/mL)	352.11 ± 40.70^*∗∗∗*^	409.48 ± 62.23^*∗∗*^	138.41 ± 13.56^*∗∗∗*^	293.74 ± 20.93^*∗∗∗*^
	AUC_(0–*∞*)_ (ng/mL)	377.75 ± 46.32^*∗∗∗*^	419.75 ± 65.31^*∗∗*^	163.60 ± 18.38^*∗∗∗*^	319.30 ± 22.66^*∗∗∗*^
	AUC_C_	271.76 ± 33.12^*∗∗∗*^	677.02 ± 105.34^*∗∗*^	53.82 ± 6.05^*∗∗*^	212.87 ± 15.10^*∗∗∗*^

Radix Ginseng Rubra	*T*_max_ (h)	0.50	0.50	0.50	0.50
	*C*_max_ (ng/mL)	80.94 ± 19.53	152.49 ± 30.98	32.82 ± 10.65	53.44 ± 20.03
	*t*_1/2_ (h)	2.94 ± 0.76	2.85 ± 0.69	3.70 ± 0.43	4.14 ± 1.18
	AUC_(0–*t*)_ (ng/mL)	85.92 ± 16.52	157.53 ± 25.46	28.98 ± 3.77	92.45 ± 10.58
	AUC_(0–*∞*)_ (ng/mL)	86.96 ± 16.89	159.27 ± 26.03	29.54 ± 4.19	95.06 ± 10.99
	AUC_C_	86.90 ± 16.89	159.20 ± 26.03	29.50 ± 4.19	95.00 ± 10.99

Mean ± SD. ^*∗*^*P* < 0.05, ^*∗∗*^*P* < 0.01, ^*∗∗∗*^*P* < 0.001 compared with those from Radix Ginseng Rubra extract.

## Abbreviations

LC-MS: Liquid chromatography-mass spectrometric method
LLOQ: Lower limit of quantification
P-gp: P-Glycoprotein
QC: Quality control
SIM: Selected ion monitoring
SMS: Sheng-Mai-San
TCM: Traditional Chinese medicine.
